# Pentraxin 3 Is Closely Associated With Tubulointerstitial Injury in Lupus Nephritis: A Large Multicenter Cross-Sectional Study

**DOI:** 10.1097/MD.0000000000002520

**Published:** 2016-01-22

**Authors:** Yun Pang, Ying Tan, Yongzhe Li, Jianchun Zhang, Yongbing Guo, Zhiling Guo, Chengying Zhang, Feng Yu, Ming-hui Zhao

**Affiliations:** From the Renal Division, Department of Medicine, Peking University First Hospital, Beijing, P.R. China (YP, YT, FY, M-HZ); Institute of Nephrology, Peking University, Beijing, P.R. China (YP, YT, FY, M-HZ); Key Laboratory of Renal Disease, Ministry of Health of China, Beijing, P.R. China (YP, YT, FY, M-HZ); Key Laboratory of CKD Prevention and Treatment, Ministry of Education of China, Beijing, P.R. China (YP, YT, FY, M-HZ); Department of Rheumatology and Clinical Immunology, Peking Union Medical College Hospital, Beijing, P.R. China (YL); Chinese Academy of Medical Sciences & Peking Union Medical College, Beijing, P.R. China (YL); Key Laboratory of Rheumatology and Clinical Immunology, Ministry of Education, Beijing, P.R. China (YL); Renal Division, Jing Dong Yu Mei Traditional Chinese Medicine and Western Medicine Integrative Kidney Disease Hospital, Hebei, P.R. China (JZ); Department of Nephrology, Anyang District Hospital, Henan, P.R. China (YG); Department of Nephrology, First Affiliated Hospital of Henan University of Science and Technology, Henan, P.R. China (ZG); Department of Nephrology, Beijing General Hospital of Armed Police Forces, Beijing, P.R. China (CZ); Department of Nephrology, Peking University International Hospital, Beijing, P.R. China (FY); and Peking-Tsinghua Center for Life Sciences, Beijing, P.R. China ( M-HZ).

## Abstract

Lupus nephritis always elicits immune inflammatory tissue damages in kidney. Pentraxin 3 (PTX3), mainly produced at inflammatory sites, is known to be involved in the regulation of the innate immunity system. The aim of this study was to investigate the serum and urine levels of PTX3, and the expression of PTX3 in renal tissues in lupus nephritis patients from a large Chinese cohort.

The study used cross-sectional survey and 288 active lupus nephritis patients, including discovery cohort and validation cohort, 115 systemic lupus erythematosus (SLE) patients without clinical renal involvement and 46 healthy controls were enrolled. Serum and urine PTX3 were screened by enzyme-linked immunosorbent assay (ELISA). The renal deposition of PTX3 was detected by immunohistochemistry and immunofluorescence.

The average level of serum PTX3 in the discovery cohort of lupus nephritis was significantly higher than that in nonrenal involvement SLE group and normal controls (*P* < 0.001, *P* < 0.001, respectively), which was confirmed by the validation cohort. Serum PTX3 levels of 15 lupus nephritis patients in remission decreased significantly compared with that in active phase. Serum PTX3 levels were significantly higher in patients with hematuria (*P* = 0.014), leucocyturia (*P* = 0.002), acute renal failure (*P* = 0.001), and nephrotic syndrome (*P* = 0.036). There were significant correlations between serum PTX3 levels and Systemic Lupus Erythematosus Disease Activity Index (SLEDAI) scores, serum creatinine value, renal pathological activity indices, and serum complement 3 (C3) in active lupus nephritis patients. The urinary PTX3 levels were significantly higher in active lupus nephritis patients compared with patients in remission and normal controls (*P* = 0.011, *P* = 0.008, respectively). There were significant associations between urinary PTX3 levels and multiple indices of tubulointerstitial lesions, including urinary KIM-1 (r = 0.368, *P* = 0.016), neutrophil gelatinase-associated lipocalin (NGAL) (r = 0.320, *P* = 0.039), microalbumin (r = 0.621, *P* = 0.003), transferring (r = 0.451, *P* = 0.040) levels and renal pathological indices scores, especially interstitial inflammation (r = 0.349, *P* = 0.025) in active lupus nephritis patients. A significant correlation was found between serum and urine PTX3 levels (r = 0.431, *P* = 0.006). PTX3 staining was mainly observed in tubulointerstitial areas of patients with lupus nephritis, and immunofluorescence study showed that PTX3 could colocalize with fibroblast in interstitium.

Circulating and local PTX3 levels were significantly increased in patients with active lupus nephritis and might be a biomarker for the disease progression, especially of tubulointerstitial injury.

## INTRODUCTION

Systemic lupus erythematosus (SLE) is a prototypical autoimmune disease, and renal involvement is an important predictor of morbidity and mortality in SLE patients.^1^ The postulated mechanisms of lupus nephritis included excessive deposition of preformed immune complexes or binding of autoantibodies to antigens localized in kidneys, which might result in immune inflammatory tissue damages.^2–4^ Moreover, identification of specific biomarkers in lupus nephritis, distinct from SLE patients without renal injury, is important for predicting renal involvement, reflecting clinical and pathological disease activity, guiding treatment, monitoring relapse and judging prognosis. However, the conventional laboratory indices such as serum creatinine value, proteinuria or serum complement 3 (C3) concentration, remained not specific and sensitive enough.^5^

Pentraxins, as a family of acute-phase proteins characterized by a cyclic multimeric structure, have been shown as markers of inflammatory response, and also involved in the regulation of the innate immunity system, including modulating the complement activation, regulating the clearance of apoptotic cells, participating in the maintenance course of immunologic tolerance, etc.^6–9^ Pentraxin 3 (PTX3), a prototypical long pentraxin, is formed by a C-terminal domain homologous to classic short pentraxins and an N-terminal 178-amino acid unrelated domain.^10^ It was originally cloned as a gene highly induced mainly by IL-1β in endothelial cells^11^ and by tumor necrosis factor-α (TNF-α) in fibroblasts.^12^ Unlike short pentraxin C-reactive protein (CRP), which is restrictedly produced in the liver, PTX3 is rapidly produced at inflammatory sites by several types of cells, including dendritic cells, macrophages, fibroblasts, endothelial cells and renal native cells, etc., upon stimulation with inflammatory factors.^13^

Increased levels of serum PTX3 have been found in a set of inflammatory autoimmune disorders, such as rheumatoid arthritis and small vessel vasculitis.^14,15^ Interestingly, limited studies found that patients with SLE presented with lower serum PTX3 levels even compared with normal controls.^15,16^ Abundance of PTX3 was found at sites of leukocytoclastic lesions in patients with small vessel vasculitis.^17^

However, lupus nephritis was characterized as local renal events at the sites of inflammation, in comparison with SLE patients without renal involvement. Thus, we speculated that the expression of PTX3 in lupus nephritis might be different and serve as a biomarker of local inflammation. This study was to investigate the circulating and local levels of PTX3 in lupus nephritis from large North Chinese multicenter cohorts, and their correlation with clinical and histopathological parameters was studied.

## MATERIALS AND METHODS

### Patients and Samples

Complete data from 213 patients with renal biopsy-proven lupus nephritis, diagnosed between 2000 and 2010 in Peking University First Hospital, were collected as the discovery cohort. Seventy-five active lupus nephritis patients were also enrolled as the validation cohort from other 4 centers, including 21 from Jing Dong Yu Mei Traditional Chinese Medicine and Western Medicine Integrative Kidney Disease Hospital, 20 from Anyang district hospital, 20 from the First Affiliated Hospital of Henan University of Science and Technology, and 14 from Beijing General Hospital of Armed Police Forces. One hundred fifteen active SLE patients with negative urinalysis (urinary protein excretion <0.5 g/day or <3+ and without any cast, eg, red cell cast, hemoglobin cast, granular cast, or mixed cast) and normal renal function (nonrenal SLE) from 2 centers, including 75 from Peking University First Hospital and 40 from Peking Union Medical College Hospital, were selected as disease controls. Forty-eight minimal change disease patients were also selected as disease controls. The SLE patients all fulfilled the 1997 American College of Rheumatology revised criteria for SLE.^18^

Sera from all the patients were obtained from peripheral blood on the day of renal biopsy or at presentation before immunosuppressive treatment in all patients in active phase. Sera from 15 patients among the 213 lupus nephritis patients in Peking University First Hospital at remission phase were also collected. Sera from 46 healthy subjects, matched for gender and age, were used as normal controls.

Urine samples from 45 of the 213 lupus nephritis patients in Peking University First Hospital were collected at the same time as sera. Urine samples of 25 lupus nephritis patients at remission were also collected. Urine samples from 30 age and sex matched healthy individuals were collected as the normal controls.

Renal biopsy samples of 35 patients randomly selected from the 213 lupus nephritis patients in Peking University First Hospital were included for the pathological study, and repeated kidney biopsies were carried out in four of them. Renal biopsy samples of IgA nephropathy and minimal change disease patients from the Peking University First Hospital were used as disease controls. Renal tissues obtained from the normal part of a nephrectomized kidney due to renal carcinoma were used as normal controls.

All the serum and urinary samples were stored at −70°C until use, and repeated freeze/thaw cycles were avoided. Informed consent was obtained for blood and urine sampling and renal biopsy from each patient. The research was in compliance with the Declaration of Helsinki. The design of this work was approved by the ethical committees of Peking University First Hospital.

### Clinical Evaluation

The following clinical data were collected and analyzed: gender, fever, malar rash, photosensitivity, oral ulcer, alopecia, arthritis, serositis, neurologic disorder, anemia, leukocytopenia, thrombocytopenia, hematuria, and leukocyturia. Clinical disease activity was assessed using the Systemic Lupus Erythematosus Disease Activity Index (SLEDAI).^19,20^

The renal response to the therapy includes complete remission, partial remission and treatment failure was detailed in previous studies.^21^ The patients were followed up in the outpatient clinic specified for lupus nephritis. The primary end point was defined as death, and secondary end points were defined as end stage renal disease or doubling of serum creatinine.

### Laboratory Assessment

Serum ANAs, anti-dsDNA antibodies, antiextractable nuclear antigen antibodies, serum C3, serum and urinary creatinine levels, urinary microalbumin, transferrin, α1-microglobulinand and *N*-acetyl-β-d-glucosaminidase (NAG), urinary kidney injury molecule-1 (KIM-1), and neutrophil gelatinase-associated lipocalin (NGAL) were all detected by commercial kits.

### Renal Histopathology

The renal biopsy specimens were examined by light microscopy, direct immunofluorescence, and electron microscopy techniques.

### Light Microscopy Examination

Renal biopsy specimens were fixed in 4.5% buffered formaldehyde for light microscopy. Consecutive serial 3-μm sections were used for histological staining. Stains employed included hematoxylin and eosin, periodic acid-Schiff, silver methenamine, and Masson trichrome.

Lupus nephritis was reclassified according to the International Society of Nephrology/Renal Pathology Society (ISN/RPS) 2003 classification system.^22^ Pathological parameters were determined by renal pathologists using a previously reported system.^23,24^

### Direct Immunofluorescence Examination

Direct immunofluorescence for IgG, IgA, IgM, C3, C1q, and fibrin deposits were semi-quantitatively graded from 0 to 4 according to the intensity of fluorescence.

### Electron Microscopy Examination

Renal biopsy specimens were fixed in 2.5% paraformaldehyde for electron microscopy. After embedded in epon, ultrathin sections were mounted on metal grids and stained with uranyl acetate before viewed in a transmission electron microscope (JEM-1230; JEOL, Tokyo, Japan).

### Detection of Serum and Urinary PTX3 Levels Using ELISA

Serum and urinary concentrations of PTX3 were both measured by a sandwich enzyme-linked immunosorbent assay (ELISA) based on the PTX3-specific monoclonal antibody MNB4 (Enzo Life Sciences, Lausen, Switzerland) and on the biotinylated rabbit PTX3-specific polyclonal IgG (donated by Humanitas Mirasole s.p.a).^25,26^ In brief, ELISA plates (Costar, Corning, NY) were coated with 2 μg/ml of monoclonal mouse anti-PTX3 antibody MNB4 diluted in bicarbonate buffer and were incubated overnight at 4°C. The plates were washed three times with PBS with 0.1% Tween 20. After blocked with 1% bovine serum albumin for 1 hour at 37°C, either recombinant human PTX3 standard or diluted samples in PBS supplemented with 1% BSA and 10 mmol/L EDTA were added and incubated for 1 hour at 37°C. After 3 washes, biotinylated polyclonal rabbit anti-PTX3 antibodies diluted to 1:2000 was added for 1 hour at 37°C. Then, horseradish peroxidase-conjugated streptavidin (Sigma, San Francisco, USA) diluted to 1:2000 was added. The reaction was developed with 3,30,5,50-tetramethylbenzidine liquid substrate system and was stopped with 1 M H_2_SO_4_. The results were recorded as the net optical absorbance at 450 and 570 nm in an ELISA reader (Bio-Rad 550, California, USA). This assay was highly sensitive and specific, and no cross-reactions were observed with other pentraxins.^26^

Serial concentrations of commercial recombinant human PTX3 standard from 0 to 100 ng/ml were used to develop standard curve. The interassay and intraassay variances were 1.78% and 4.79% in multiple repeats, which proved that the method was accurate and reliable.

### Detection of Serum Levels of TNF-α and IL-1β by ELISA

Serum levels of TNF-α and IL-1β, 2 main cytokines that induce PTX3-production, were measured using ELISA by an Enzyme Immunoassay Kit (R&D Systems, Abingdon, UK) with a sensitivity of 0.1 ng/ml, according to the recommendations from the manufacturer.

### Detection of Renal Expression of PTX3 by Immunohistochemistry

Immunohistochemical staining of PTX3 was performed on 4-μm deparaffinized sections of formaldehyde-fixed renal tissue using rat monoclonal antibody MNB4 as described previously.^25^ After 30 minutes in xylol, sections were rehydrated using alcohol solutions. After microwave treatment for antigen retrieval, sections were immersed into freshly prepared 3% hydrogen peroxide for 30 minutes at room temperature to quench endogenous peroxidase activity. To block nonspecific staining, sections were incubated with 3% bovine serum albumin in PBS at room temperature for 30 minutes. Then MNB4 (diluted at 1:500) were added on each section after the removal of blocking bovine serum albumin without washing and incubated overnight at 4°C. The secondary antibodies from the detection system, Dako EnVision horseradish peroxidase (Dako A/S, Copenhagen, Denmark), were incubated for 30 minutes at 37°C. Next, sections were developed in fresh hydrogen peroxide plus 3,3′, 5,5′-Tetramethylbenzidine solution for 2 minutes. Finally, the sections were incubated with hematoxylin, dehydrated through alcohols and xylene. As a negative control, primary antibodies were replaced by PBS.

The sections were examined by light microscopy. The renal staining of PTX3 was evaluated by the Image Pro Plus analysis software 6.0 (Media Cybernetics, Silver Spring, MD). The positive signals were quantified as the mean optical density (integrated option density/area).

### Colocalization of PTX3 and Cells in Kidney by Immunofluorescence

In a similar manner to immunohistochemistry, fresh frozen sections of acetone-fixed renal tissue were stained with rat monoclonal antibody MNB4 for PTX3, commercially available anti-CD68 rabbit polyclonal antibodies (Santa Cruz, California, USA) for macrophages, anti-CD31 (platelet endothelial cell adhesion molecule 1; Abcam, Cambridge, UK) for endothelial cells, anti-E-cadherin (Santa Cruz) for renal tubular epithelial cells and anti-α-smooth muscle actin (Sigma) for fibroblasts in interstitium area. For immunofluorescence analysis, tissue sections were blocked with normal donkey serum, then incubated with MNB4 and different cell markers, and revealed with fluorescein-labeled antibodies. Samples stained without primary antibodies were used as negative controls. Sections were stored shortly at 4°C before being examined using a confocal microscope (Olympus Viewer 1000, Tokyo, Japan).

### Statistical Analysis

Statistical analysis was performed with statistical software SPSS 13.0 (SPSS, Chicago, IL). Quantitative data were expressed as mean ± SD, or median with range (minimum, maximum). To compensate for differences in urine flow rate, urinary concentration of biomarkers and PTX3 were normalized for millimoles of urinary creatinine. Differences of quantitative parameters between groups were assessed using the *t* test (for normally distributed data) or nonparametric test (for nonnormally distributed data). The relationships between 2 continuous variables were analyzed using Spearman rank correlation. Multivariable linear regression analysis of PTX3 levels and renal pathological scores was performed. The Cox regression model was applied to identify prognostic factors. *P* < 0.05 was considered statistically significant.

## RESULTS

### General Data of Patients and Controls

The general data of the discovery cohort of patients with lupus nephritis is shown in Table 1. Gender and age of patients were comparable between the discovery cohort of lupus nephritis (male/female: 33/180, 32.8 ± 11.2 years), the validation cohort of lupus nephritis (male/female: 11/64, 31.4 ± 13.6 years), SLE without renal involvement group (male/female: 18/115, 29.9 ± 14.5 years), minimal change disease group (male/female: 9/39, 28.3 ± 14.8 years), and normal controls (male/female: 8/38, 36.4 ± 7.0 years) (*P* = 0.825, *P* = 0.217, respectively). The SLEDAI scores was comparable between the discovery cohort, in which the renal involvement scores were subtracted, and SLE without renal involvement group (5.3 ± 4.9 vs. 4.8 ± 5.1, *P* = 0.784).

**TABLE 1:**
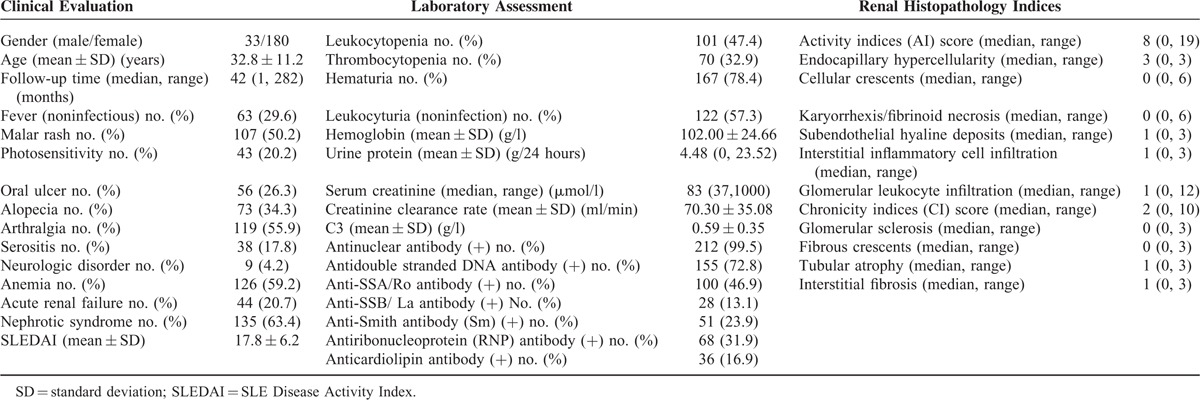
General Clinical Data of Lupus Nephritis Patients in the Discovery Cohort

### Serum PTX3 Levels in All Patients and Controls

**FIGURE 1:**
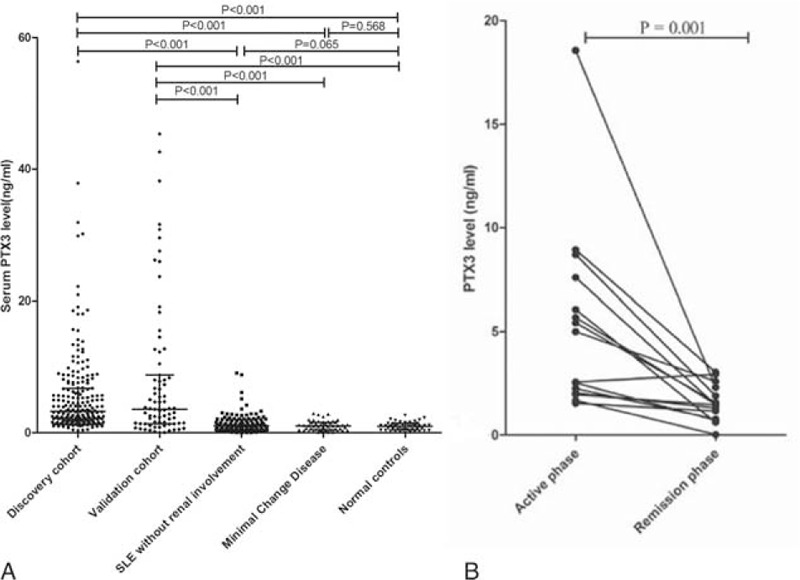
Serum PTX3 levels in different groups. (A) Serum PTX3 in patients with active lupus nephritis from discovery cohort, validation cohort, SLE without renal involvement, minimal change disease and normal controls. (B) Serum PTX3 levels in patients with lupus nephritis in active and remission phase.

The average level of serum PTX3 in the discovery cohort of lupus nephritis was significantly higher than that in SLE without renal involvement group (3.270 (0.349–56.376) vs. 1.065 (0.040–9.098) ng/ml, *P* < 0.001), minimal change disease group (3.270 (0.349–56.376) vs. 1.021 (0.105–2.940) ng/ml, *P* < 0.001), and normal controls (3.270 (0.349–56.376) vs. 0.993 (0.007–2.655) ng/ml, *P* < 0.001) (Figure 1A). These results were confirmed by the validation cohort, in which serum PTX3 levels were also significantly higher than that in SLE without renal involvement group (3.589 (0.007–45.364) vs. 1.065 (0.040–9.098) ng/ml, *P* < 0.001), minimal change disease group (3.589 (0.007–45.364) vs. 1.021 (0.105–2.940) ng/ml, *P* < 0.001) and normal controls (3.589 (0.007–45.364) vs. 0.993 (0.007–2.655) ng/ml, *P* < 0.001) (Figure 1A). There was no significant difference in the average serum PTX3 levels between SLE without renal involvement group and normal controls (*P* = 0.065).

The PTX3 levels of all the 15 patients in remission decreased significantly compared with the self-controls in active phase (1.566 ± 0.904 vs. 5.371 ± 4.637 ng/ml, *P* = 0.001) (Figure 1B).

### Association Between Serum PTX3 Levels and Clinicopathologic Data of Patients in Discovery Cohort

There was no significant difference of serum PTX3 levels between female and male in lupus nephritis patients (*P* = 0.328). There was no association between serum PTX3 level and age (r = −0.038, *P* = 0.584).

In clinical data, serum PTX3 levels were significantly higher in patients with the following clinical manifestations, including noninfectious leucocyturia (*P* = 0.002), acute renal failure (*P* = 0.001), and nephrotic syndrome (*P* = 0.036). There was a positive correlation between serum PTX3 levels and SLEDAI scores (r = 0.216, *P* = 0.002). In laboratory findings, there were significant associations between serum PTX3 levels and serum C3 (r = −0.206, *P* = 0.003) and serum creatinine (r = 0.260, *P* < 0.001).

In renal pathological data, the levels of serum PTX3 in different pathological classes of lupus nephritis were as followings: 2.899 (0.953–15.533) ng/ml in class II, 2.505 (0.382–13.311) ng/ml in class III, 4.168 (0.355–56.376) ng/ml in class IV and 2.366 (0.399–11.082) ng/ml in class V. There were significant differences in serum PTX3 levels among various pathological classes, which was the highest in class IV group (*P* = 0.009). There were significant correlations between serum PTX3 levels and renal pathological activity indices scores (r = 0.248, *P* < 0.001), endocapillary hypercellularity (r = 0.246, *P* = 0.001), and leukocyte infiltration (r = −0.191, *P* = 0.009) in active lupus nephritis patients (Table 2).

**TABLE 2:**
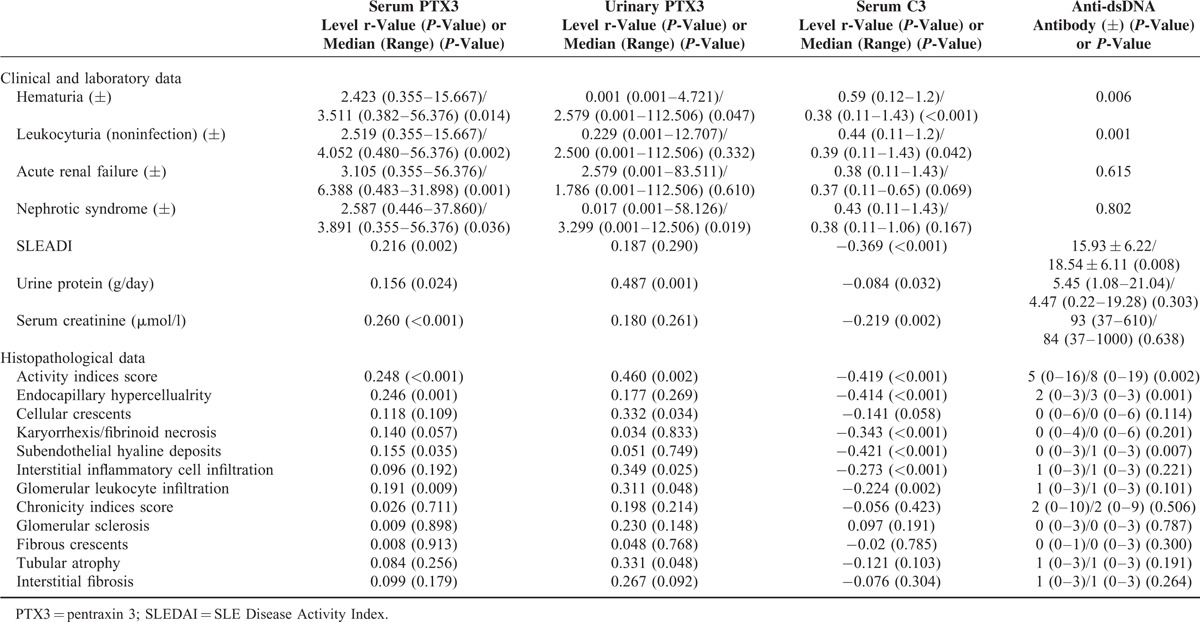
Associations Between Serum PTX3, Urinary PTX3, Serum C3, Anti-ds-DNA Antibody, and Renal Injury Indices in Patients With Lupus Nephritis

Furthermore, we did comparisons of associations between renal injury indices and serum PTX3 level and other 2 conventional biomarkers, anti-ds-DNA antibody and C3 level, in lupus nephritis. We found that the differences of some indices, including leukocyturia (noninfection), acute renal failure, nephrotic syndrome, urine protein, and serum creatinine value, were more significant in PTX3 group than the other 2 groups (details in Table 2).

The patients with lupus nephritis were followed up with a duration of 42 (1–282) months. Level of serum PTX3 was not found to be a risk factor for long-term renal outcomes in lupus nephritis using the log-rank test for univariate survival analysis of renal prognosis (*P* = 0.594, HR = 0.967, 95% CI: 0.859–1.095).

### Serum Levels of TNF-α and IL-1β in Discovery Cohort and Controls

TNF-α and IL-1β were considered as 2 main cytokines which induced the production of PTX3. Thus, we further detected serum levels of TNF-α and IL-1β in active lupus nephritis patients and normal controls.

Lupus nephritis group had significantly higher levels of TNF-α and IL-1β compared to healthy controls (5.967 ± 2.402 ng/ml vs. 5.098 ± 1.072 ng/ml, *P* = 0.023; 6.213 ± 2.807 ng/ml vs. 4.441 ± 1.381 ng/ml, *P* = 0.001, respectively). Moreover, in patients with lupus nephritis, a significant correlation was found between serum TNF-α concentration and serum PTX3 level (r = 0.351, *P* = 0.012), but not between IL-1β and PTX3 (r = 0.062, *P* = 0.861).

### Urinary PTX3 Levels in Active Lupus Nephritis Patients and Their Clinicopathologic Associations

**FIGURE 2:**
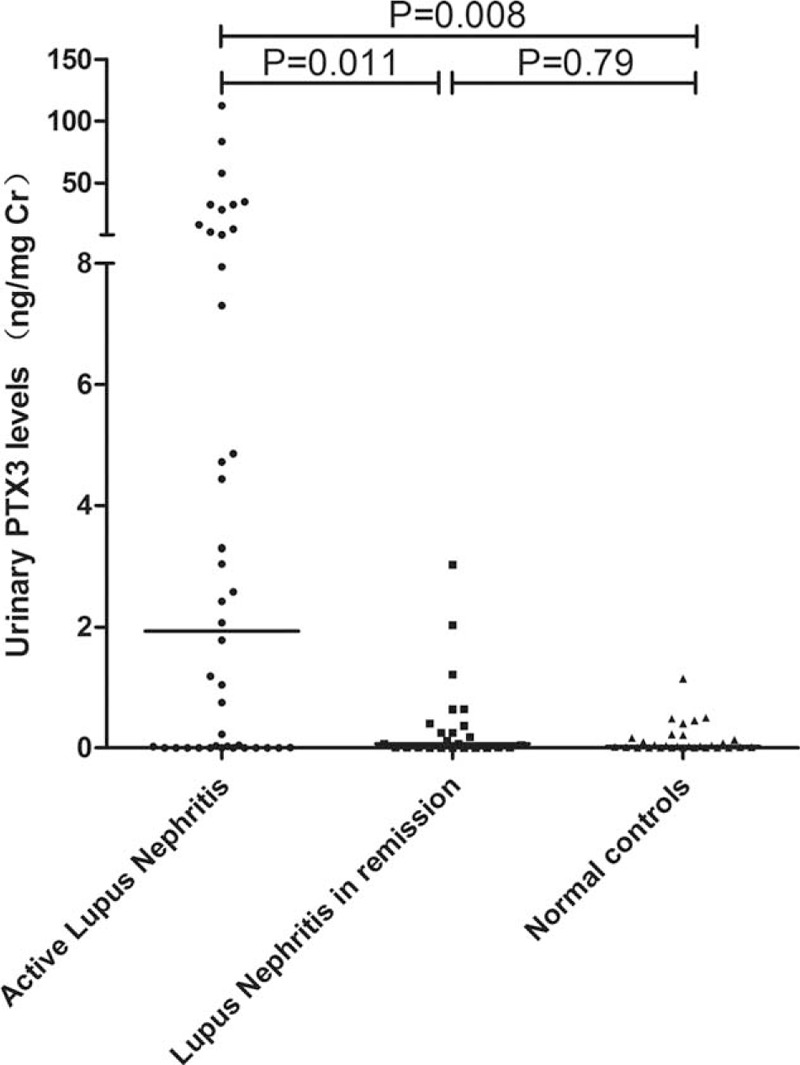
Urinary PTX3 levels in different groups: Patients with active lupus nephritis, patients with lupus nephritis in remission and normal controls.

The urinary PTX3 levels were significantly higher in active lupus nephritis group compared with lupus nephritis patients in remission and normal controls (1.929 (0.001–112.506) vs. 0.068 (0.001–3.031) ng/mg Cr, *P* = 0.011; 1.929 (0.001–112.506) vs. 0.023 (0.002–1.143) ng/mg Cr, *P* = 0.008). No significant difference was found in urinary levels of PTX3 between lupus nephritis patients in remission and normal controls (0.068 (0.001–3.031) vs. 0.023 (0.002–1.143) ng/mg Cr, *P* = 0.79) (Figure 2).

There was no significant difference of urinary PTX3 levels between female and male in lupus nephritis patients (*P* = 0.577). There was no association between urinary PTX3 level and age (r = −0.288, *P* = 0.068).

In clinical data, the urinary PTX3 levels were significantly higher in patients with hematuria (*P* = 0.047) and nephrotic syndrome (*P* = 0.019). In laboratory findings, there were significant associations between urinary PTX3 levels and the following renal injury indices: urine protein (r = 0.487, *P* = 0.001), urinary KIM-1 (r = 0.368, *P* = 0.016), urinary NGAL (r = 0.320, *P* = 0.039), urinary microalbumin (r = 0.621, *P* = 0.003), and urinary transferrin (r = 0.451, *P* = 0.040).

Correlation analysis showed that there were significant correlations between urinary PTX3 levels and renal pathological activity indices scores (r = 0.460, *P* = 0.002), cellular crescents (r = 0.332, *P* = 0.034), interstitial inflammation (r = 0.349, *P* = 0.025), leukocyte infiltration (r = 0.311, *P* = 0.048), and tubular atrophy (r = 0.311, *P* = 0.048) in active lupus nephritis patients (Table 2). The multiple linear regression analysis showed that interstitial inflammation was the main effect factor of urinary PTX3 levels among all the renal pathological parameters (β = 0.497, *P* = 0.001).

We also found that the differences of nephrotic syndrome, proteinuria, activity indices score, cellular crescents, interstitial inflammation cell infiltration, leukocyte infiltration and tubular atrophy, were more significant in PTX3 group than anti-ds-DNA antibody or C3 level groups (details in Table 2).

There were significant correlations between urinary and serum PTX3 levels in patients with lupus nephritis (r = 0.431, *P* = 0.006).

### Expression of PTX3 in Renal Tissues in Active Lupus Nephritis Patients and Its Pathological Associations

**FIGURE 3:**
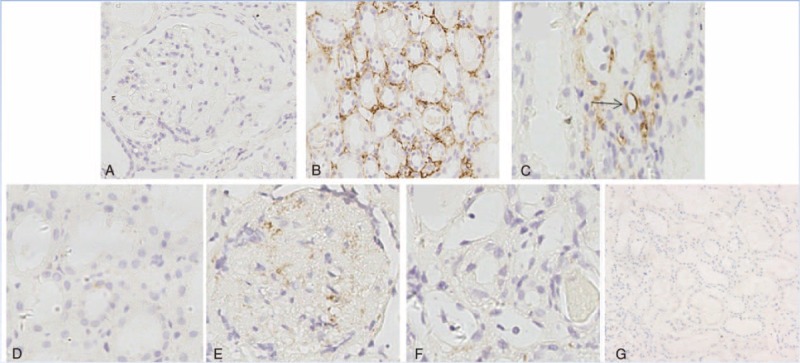
Immunohistochemistry staining for PTX3 in renal specimens of patients with active LN controls (original magnification ×400): (A–C) PTX3 staining in active lupus nephritis patients (the arrow indicating the small vessel). (D) PTX3 staining in lupus nephritis patient with the omission of the PTX3-specific primary antibody. (E) PTX3 staining in IgA nephropathy patients. (F) PTX3 staining in minimal change disease patient. (G) PTX3 staining in normal control.

The PTX3 expressions in renal tissues were investigated in 35 patients with active lupus nephritis by immunohistochemistry. The PTX3 staining was absent in glomeruli despite the proliferative and inflammatory lesions (Figure 3A). Intense staining for PTX3 was mainly observed in the tubulointerstitial areas of lupus nephritis patients (Figure 3B). In some patients, staining could also be found in small vessels (Figure 3C). The omission of the PTX3-specific primary antibody completely abolished the staining (Figure 3D). PTX3 could be detected in the mesangium of IgA nephropathy patients (Figure 3E), which was consistent with previous study.^27^ Renal tissues from minimal change disease patients and normal control stained negative for PTX3 (Figure 3F and G). The mean optical density of PTX3 in tubulointerstitium of lupus nephritis was 0.46 ± 0.28 and correlated positively with total renal activity indices score (r = 0.372, *P* = 0.021), interstitial inflammation (r = 0.369, *P* = 0.010), and interstitial fibrosis (r = 0.326, *P* = 0.038) in lupus nephritis group, respectively.

Repeated kidney biopsies were carried out in 4 of the 35 lupus nephritis patients. The mean optical density of PTX3 changed accordingly with disease activity, especially the degree of renal pathological damage. In Patient 1, the mean optical density of PTX3 increased at the disease relapsing phase, and the mean optical density of PTX3 decreased at the remission phase in Patients 2, 3, and 4 (Table 3).

**TABLE 3:**
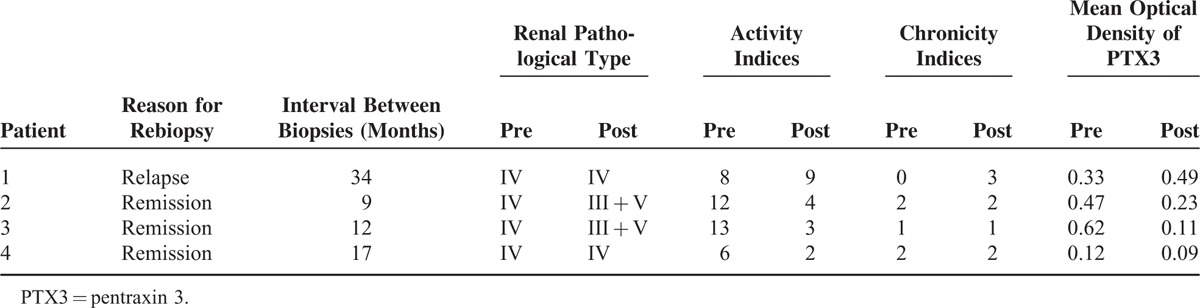
Profiles of Lupus Nephritis Patients Who Underwent Repeated Biopsies

No correlation was found between the mean optical density of PTX3 in renal specimens and the serum or urinary levels of PTX3 in lupus nephritis.

### Identification of Cellular Source of PTX3 in Renal Tissues From Patients With Lupus Nephritis

In order to identify the cell types that contribute to the production of PTX3 in lupus nephritis, kidney biopsies were double stained for several markers of renal cells and PTX3 by immunofluorescence.

**FIGURE 4:**
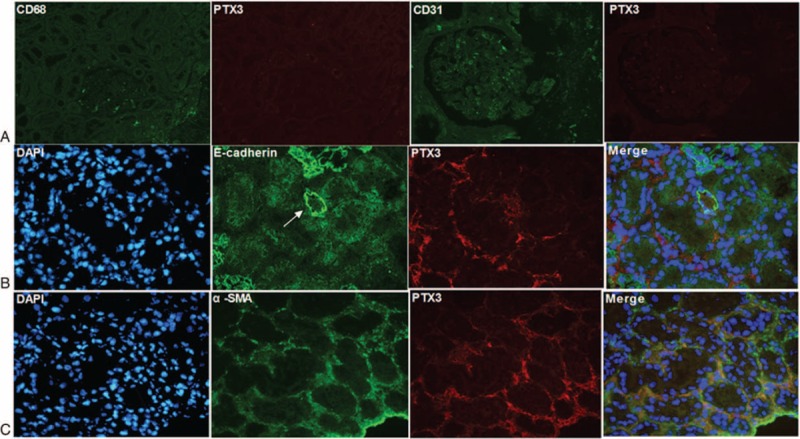
Colocalization of PTX3 and renal cells markers on kidney sections of patients with active LN (original magnification ×400): (A) The left 2 photos show double staining of CD68 and PTX3, and they failed to be colocalized; The right 2 photos show double staining of CD31 and PTX3. Glomerular endothelial cell, positive for CD31, also showed no expression of PTX3. (B) Double staining of tubular epithelial cell maker, E-cadherin and PTX3 showed no colocation (the arrow indicating the renal tubule). (C) PTX3 and fibroblast marker, α-SMA, colocalized in interstitium, merged in yellow.

As shown in Figure 4A, CD68, the macrophage cell marker, and PTX3 failed to be colocalized. Glomerular endothelial cell, represented by CD31, also showed no expression of PTX3 (Figure 4A). Double staining for the fibroblast marker, α-smooth muscle actin (α-SMA), and PTX3 showed that PTX3 was mainly colocalized with fibroblast in interstitium, instead of tubular epithelial cell, represented by E-cadherin (Figure 4B and C). The omission of the PTX3-specific primary antibody completely abolished the labeling.

## DISCUSSION

In the present study, for the first time using large multicenter cohorts, including the discovery cohort and validation cohort, we found that the serum levels of PTX3 increased significantly in active lupus nephritis patients compared with healthy controls, but not in SLE without renal involvement group although with comparable SLEDAI. It was consistent with the previous small sample studies.^16,17,28^ Significant correlations were found between serum PTX3 levels and some clinical active indices of lupus nephritis, including hematuria, noninfectious leucocyturia, serum creatinine value, serum C3 level, and SLEDAI scores. Furthermore, in renal histopathology evaluation, serum PTX3 levels were the highest in patients with class IV lupus nephritis. Serum PTX3 levels positively correlated with total activity indices, endocapillary hypercellularity, and leukocyte infiltration. More importantly, serum PTX3 levels decreased significantly in the remission phase. These results suggested that serum PTX3 level was closely associated with the disease activity and severity of lupus nephritis, which was different with SLE without renal involvement. Thus, we raised the possibility that renal locally produced PTX3 might be involved in the progression of lupus nephritis.

To verify the above hypothesis, we further detected the urinary PTX3 levels and intrarenal PTX3 expression in lupus nephritis patients. Urinary PTX3 levels were found elevate significantly in patients with active lupus nephritis than that in remission and normal controls. Further correlative analysis indicated that, besides its significant associations with proteinuria like serum PTX3, especially there were strong positive associations between urinary PTX3 levels and urinary microalbumin, transferring, KIM-1 and NGAL levels, which were sensitive biomarkers of acute renal injury and acute tubular-interstitial injury.^29,30^ Relatively, urinary PTX3 levels were also correlated with some renal pathological indices, in which interstitial inflammation was the strongest one. The differences of interstitial inflammation cell infiltration and tubular atrophy were more significant in PTX3 group than anti-ds-DNA antibody and C3 level groups.

Next, the immunohistochemistry results showed that PTX3 were mainly found in tubulointerstitial areas of lupus nephritis patients but negative in glomeruli, in comparison with it stained positive in the mesangium of IgA nephropathy patients which was consistent with Bussolati report.^27^ The mean optical density of PTX3 in tubulointerstitium correlated closely with the extent of renal activity indices such as interstitial inflammation and interstitial fibrosis in lupus nephritis. Moreover, staining on repeated kidney biopsies showed that the mean optical density of PTX3 changed accordingly with the degree of renal pathological damage. Consistently, double staining by immunofluorescence suggested that PTX3 was expressed mainly by fibroblast in interstitium. Interestingly, we also found that there was a significantly positive correlation between serum PTX3 and TNF-α level, the major cytokine induced PTX3 production from fibroblast.^12^ These results supported that PTX3 expression in the kidneys correlated with clinicohistopathological injury indices, especially tubular-interstitial features, which might result from the persistence of the major source, renal interstitial fibroblasts, and spill into the urine or serum, in active lupus nephritis. However, the absence of glomerular PTX3 expression despite the presence of inflammatory cells in glomeruli is puzzling. Interestingly, a recent experiment showed that transient kidney injury in lupus prone hosts could induce some inflammatory cytokine, such as colony stimulating factor 1 (CSF-1), which in turn leads to macrophage-mediated tubulointerstitial, followed by a rise in autoantibodies and immune complex-mediated glomerular nephritis.^31^ Thus, it could be inferred that PTX3 related interstitial inflammation precedes and perhaps triggers glomerular disease in lupus nephritis, which needs further studies.

Several studies, including ours, indicated that the tubulointerstitial lesions were high risk factors for renal outcomes of lupus nephritis,^32,33^ and its presence might adversely affect the prognosis of the patients.^23,34^ Thus, tracking specific and sensitive biomarkers of tubulointerstitial injury is important for monitoring activity, guiding treatment, and judging prognosis in lupus nephritis patients, though the related studies were limited.^35,36^ In this study, it showed that circulating and local PTX3 might be a biomarker for disease activity of lupus nephritis, especially for the tubulointerstitial injury. Due to the few end-points in our study, we could not analyze the status of urinary PTX3 in long-term renal outcomes, which needs further explorations with more patients and longer observational time.

Though a molecule as a biomarker might not participate in the pathogenesis of disease, we believed that PTX3 should be involved in the progression of lupus nephritis based on the following reasons: As having multiple complex nonredundant functions, PTX3 was mainly involved in the regulation of inflammatory reactions and the maintenance of immunologic tolerance, which were 2 important fields in the mechanism of lupus nephritis. The pathogenesis of tubulointerstitial inflammation in lupus nephritis is not entirely clear. Previous studies suggested that exposed to anti-dsDNA antibodies or other stimulators of lupus, tubular epithelial cells play a central role in tubulointerstitial inflammation via cross-talk with inflammatory cells by production of a variety of inflammatory mediators in lupus nephritis, including IL-6, monocyte chemotactic protein-1, soluble interleukin 7, CSF-1and TNF-α, which could sustained renal inflammation.^36–38^ NF-κB activation pathway and tubular Toll-like receptor 9 (TLR-9) activation were demonstrated to be involved in this process.^39,40^ Under the environment of lupus, PTX3 might be upregulated in fibroblasts during early phase and play a role in regulating tubulointerstitial inflammation loci. More interestingly, recent researches found that tubulointerstitial inflammation in human lupus nephritis was associated with in situ adaptive immunity. Tertiary lymphoid organ-like structures are common in tubulointerstitial inflammation and in situ antigen-driven selection of B cells that locally secrete pathogenic antibodies could occur in each of these structures.^41^ Lately, Kinloch et al^42^ identified vimentin as the dominant autoantigen driving in situ adaptive immunity in lupus tubulointerstitial nephritis. Several studies have indicated that PTX3 could regulate antigen presenting cells to take up and present apoptotic cells.^43,44^ Could PTX3 participate in the local B cell expansion or the presenting antigens to coresident T cells by resident B cells in the interstitium? Or whether abundant PTX3 could locally prime antigen presenting cells and become antigenicity? As PTX3 could be both an enhancement and inhibition of the inflammatory process depending on the way which it regulated the complement activation,^45,46^ the “double edged roles” of PTX3 on tubulointerstitial inflammation and the signaling mechanisms underlying this in lupus nephritis need further studies.

In conclusion, our study showed that the significantly increased circulating and local PTX3 levels could be found in patients with active lupus nephritis and PTX3 might be a biomarker for the disease progression, especially tubulointerstitial injury. The exact role of PTX3 in the pathogenesis of lupus nephritis needs further explorations.
